# Round about the Asylums—II

**Published:** 1888-03-17

**Authors:** 


### Round About the Asylums.—II.

By a Medical Superintendent.

### EAST RIDING ASYLUM.

This is the sixteenth annual report of the asylum at Beverley, which has the merit, or otherwise, of being the smallest county asylum in England. It contains but 268 patients, and 35 of these are private cases. The charge to the unions is 8*s.* 9*d.* a week—a rate which must be looked on as extremely low when the size of the asylum is remembered. The private patients pay from 13*s.* to 30*s.* Unless the section of the Act which requires private patients in county asylums to have in all respects the same accommodation as paupers be broken, the last-named charge would seem to be too high.

Several improvements have been carried out during the year; among others a new committee-room has been built at a cost of £420. It ought to be a good one. Iron staircases are being fitted up to some of the dormitories, so that in the event of fire ready means of exit may be at hand.

The visitors state that the management of the asylum under Dr. Macleod has been perfectly satisfactory. The medical superintendent remarks, in his report, that "there is an absence of the more acute forms of the disease, and the insanity most prevalent strikes me as a mere exaggeration of a general somewhat low standard of intelligence." This is very likely true; but isn't it a little "rough" on the East Riding?

The recovery-rate stands at 40 per cent. on the admissions, and the death-rate at 10 per cent. on the average number resident. The first is a creditable percentage, and the second cannot be looked on as high. Additional night attendants have been appointed, and altogether the asylum would seem to be in a sound state and carefully managed. The farm accounts show a balance of £487 on the right side; but we notice beef is allowed for at 6¼*d.* per pound and milk at 11*d.* per gallon. A foot-note is also appended to the effect that the "no-rent manifesto" has been adopted, in so far as no rent is charged in the farm accounts. Thus is solved the difficult problem of farming at a profit. East Riding farmers, be careful! "No rent" will not do in England yet!

### LEICESTER AND RUTLAND COUNTY ASYLUM.

This is an old friend, or at least a middle-aged one, for it has passed its fifty-first birthday, and now issues its thirty-ninth annual report. It is a county asylum, with a charity tacked on to it. It contains 450 patients and is quite full; indeed, many of the Leicester cases are now boarded out in other asylums. The recoveries are nearly 39 per cent. on the admissions for the year, and the deaths were under 10 per cent. on the average number resident, notwithstanding that consumption seems to have been rather prevalent. Dr. Higgins states that the recoveries since the opening of the asylum have averaged 46 per cent. on the admissions. We doubt whether many English asylums can beat this record.

Dr. Higgins is not to be envied, at least as far as the class of his patients is concerned. He has 60 epileptics, 60 idiots, 20 deaf patients, 4 blind men, and 13 dumb ones. In the incurable insane, perhaps he might not think dumbness always an objection.

Not a few minor improvements have been carried out during the year, and the Commissioners say that the state of the asylum and patients is creditable to those in charge of them. They recommend the erection of a detached chapel. The weekly rate to the unions is 8*s.* 6*d.*, and some of the charity cases pay as low as 2*s.* 6*d.*

### PORTSMOUTH BOROUGH ASYLUM.

This asylum was opened in 1879 or 1880, and it then contained 452 patients. It has now 535; but 170 of these are out-county and private cases, most of the former being chargeable to the metropolitan districts. The recoveries amount to 38 per cent. on the admissions, and the deaths are a trifle over 9 per cent. on the average number resident. Seventeen of the deaths were from general paralysis and 10 from consumption. Post-mortem examinations were made in 48 out of the 50 deaths. This speaks well for the medical staff, or for the good sense of the patients' friends, or perhaps for both. The Commissioners remark that the numbers employed ought to be increased; but with such a proportion of out-county and private patients, we should feel inclined to doubt whether much can be done in this direction. The commissioners add that "the asylum was in good order, and the wards bright and cheerful."

The salaries and wages are given at page 51. We notice that the medical superintendent receives only £600 a year—a sum which cannot be thought excessive, considering the number of patients and his length of service. The weekly cost of maintenance is 10*s.* 2*d.* We do not notice the charge for out-county or private patients anywhere given.

### GLASGOW ROYAL ASYLUM.

This institution contains 470 patients, being 10 fewer than at the close of 1886. The private patients number 286, and the paupers 184. The private cases are received at rates varying from 15*s.* a week to £6 6*s.* At present there are 120 patients who pay £40 a year or under, a fact which proves that the asylum is really doing good work in relieving a class for which in England the accommodation is totally inadequate. The paupers pay from 11*s.* 8*d.* to 13*s.* 1*d.*, according to the parishes from which they are sent. We nowhere found a statement of the cost per head per week. The omission is to be regretted, as it would have been interesting to compare the cost with some of the English hospitals for the insane. The recovery-rate is very high and the death-rate very low.

It very often happens that patients are paupers for no other reason than that they are insane, and Dr. Yellowlees attributes the falling off in the number of the private admissions which took place during the year to commercial depression, the deficit going doubtless to swell the number of paupers either in his own or other asylums. He rightly deplores the fact that relief cannot be given in time to deserving cases, and yet it is difficult to see how the Poor Law could be stretched to meet them. Dr. Yellowlees adds, "It seems a bitter return for their carefulness and sobriety, that these very virtues should thus deprive them of the help which a drunken and penniless neighbour would receive at once."

Many of the Scotch superintendents will not approve of the remarks made by their *confrère* on the open-door system, and we fancy they will not go unchallenged. We are inclined to think there is something in the system when it is carried out with due care and restrictions. Non-restraint met with just the same reception in most quarters, and yet its success along the whole line has been undoubted.

The committee of the Glasgow Asylum knows how to reward faithful service. The medical superintendent receives £1,200 a year and the usual perquisites. The senior assistant gets £200 a year. The late superintendent is in receipt of £600 a year as pension.

The farm seems to have produced a profit of £380. It does not appear whether or not rent is charged.

